# Dalbavancin as a Viable Alternative for Addressing Treatment Adherence in Methicillin-Resistant Staphylococcus aureus (MRSA) Bacteremia: A Case Report

**DOI:** 10.7759/cureus.54078

**Published:** 2024-02-12

**Authors:** Michael Sabina, Jessica Cobian, Imad Dibo

**Affiliations:** 1 Internal Medicine, Lakeland Regional Health Medical Center, Lakeland, USA; 2 Pharmacy, Lakeland Regional Health, Lakeland, USA; 3 Infectious Diseases, Lakeland Regional Health Medical Center, Lakeland, USA

**Keywords:** infectious disease medicine, drug compliance, osteomyelitis, methicillin resistant staphylococcus aureus bacteremia, dalbavancin

## Abstract

Outpatient parenteral antibiotic therapy (OPAT) aims to facilitate antibiotic treatment in non-hospital settings while minimizing hospitalization costs and infection risks. However, ensuring patient adherence, especially among socially disadvantaged groups like the homeless may be challenging. Studies indicate that increased dosing frequency negatively impacts adherence rates. Simplified dosing schedules and addressing transportation barriers are protective measures.

Dalbavancin, a weekly oral antibiotic with an extended half-life, may offer a solution for methicillin-resistant Staphylococcus aureus (MRSA) bacteremia in patients with adherence challenges. We present a case of a homeless patient with MRSA bacteremia and osteomyelitis successfully treated with dalbavancin. Our case highlights the potential of dalbavancin's once-weekly dosing to improve adherence and treatment outcomes in challenging outpatient scenarios.

## Introduction

The primary objective of outpatient parenteral antibiotic therapy (OPAT) is to facilitate the completion of antibiotic treatment in non-hospital settings such as the patient's home. This approach not only aims to maintain normalcy in the patient's life but also reduces hospitalization costs and the risk of acquiring hospital-based infections [1,2]. However, a significant challenge in the effectiveness of OPAT is ensuring patient adherence to antibiotic regimens, particularly among socially disadvantaged groups, including the homeless who may lack transportation and face other barriers.

Studies have identified that increased dosing frequency adversely affects outpatient oral antibiotic adherence. Adherence rates significantly drop from 80% for once-daily dosing to 38% for three-times-daily dosing [3,4]. Protective measures against nonadherence include simplified, less frequent dosing schedules, which have been shown to improve adherence, especially in vulnerable populations [5]. Moreover, the lack of transportation emerges as a critical barrier, preventing regular follow-up visits and consistent antibiotic administration [5].

Additionally, preferences play a crucial role in adherence, with patients more likely to follow a treatment plan that aligns with their preferences [6]. This is particularly pertinent for populations with complex social issues such as those with a history of intravenous drug use or homelessness [7]. These groups are more prone to complications like lost to follow-up, secondary infections, and higher readmission rates, underlining the need for more tailored antibiotic regimens. Currently, the Infectious Diseases Society of America guidelines recommend a once-daily intravenous dose of vancomycin dosed at 6 mg/kg for a minimum of eight weeks [8].

In this context, dalbavancin, a semisynthetic glycopeptide antibiotic, represents a promising alternative to traditional glycopeptides such as vancomycin. Dalbavancin acts by forming a complex with the C-terminal D-alanyl-D-alanine of growing peptidoglycan chains in bacterial cell walls, thereby inhibiting cell wall biosynthesis [9]. It is effective against resistant strains like methicillin-resistant Staphylococcus aureus (MRSA). Approved for acute bacterial skin and skin structure infections, it is administered as weekly oral dosing. Its extended half-life and favorable safety profile make it a potential off-label option for MRSA bacteremia in patients facing adherence challenges, including the homeless and those without regular access to healthcare facilities.

## Case presentation

A middle-aged male with a history of homelessness, intravenous drug abuse, and previously managed tibial fracture, with open reduction and internal fixation (ORIF) and hardware placement, presented to the emergency department (ED) with complaints of pustular drainage from a tibial wound. This wound was sustained several weeks prior in a motor vehicle collision while the patient was riding a bicycle. The patient presented with signs of systemic illness, such as fever, chills, tachycardia, and elevated lactic acid. Laboratory values were significant for markedly elevated leukocytes and erythrocyte sedimentation rate (ESR).

**Figure 1:**
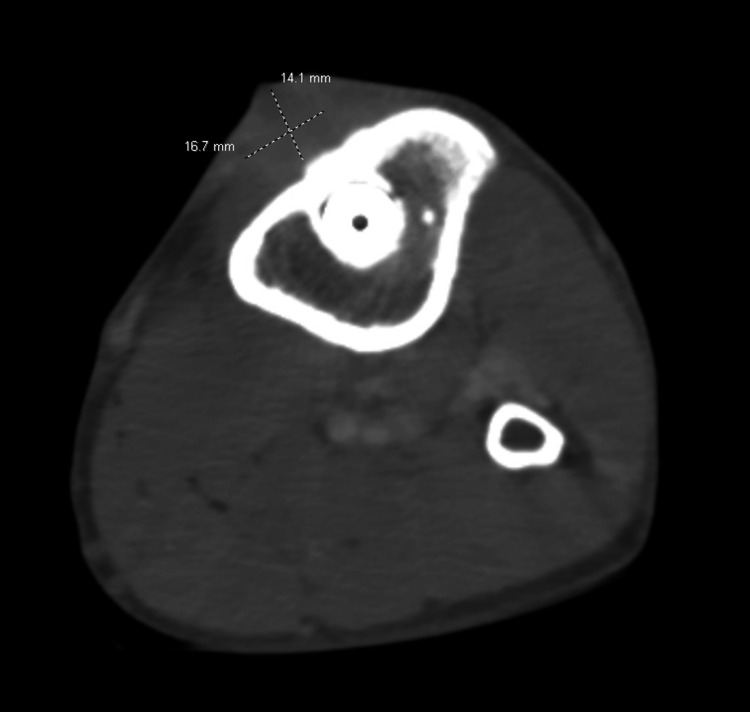
Computed tomography (CT) scan of the left lower extremity

The computed tomography (CT) scan of the left lower extremity revealed an abnormal bony lucency surrounding the distal screw within the proximal left tibia, which extended through the anterior bony cortex of the left tibia (Figure 1). Findings were suspicious for osteomyelitis. There was also notable soft tissue swelling with enhancing fluid collection overlying this area, suspicious for an abscess collection.

Wound cultures from the affected site tested positive for MRSA (Table 1), and subsequent blood cultures also revealed MRSA bacteremia.

**Table 1:** Deep wound culture results with sensitivities

	Methicillin-resistant Staphylococcus aureus	
Drug	MIC Interp	MIC Dilutn
Clindamycin	R	>=8
Erythromycin	R	>=8
Gentamicin	S	<=0.5
Oxacillin	R	>=4
Penicillin	R	>=0.5
Tetracycline	R	>=16
Trimethoprim/Sulfa	R	>=320
Vancomycin	S	1

The patient underwent successful surgical removal of the hardware and was initiated on intravenous vancomycin. After 72 hours of parenteral antibiotic treatment, blood cultures were negative for MRSA, indicating a response to therapy. Despite initial treatment, concerns arose regarding the patient's adherence to an outpatient regimen that would require a peripherally inserted central catheter (PICC) line placement and daily visits to our fast-track unit for IV vancomycin administration for a total of six weeks. The concerns were largely due to his social circumstances of homelessness, no transportation, history of IV drug use, and ultimately a low likelihood of him adhering to the treatment plan. A decision was made to administer an off-label loading dose of dalbavancin (1500 mg), followed by a second dose eight days post-discharge. The patient was also prescribed linezolid 600 milligrams twice a day, to be commenced two weeks after the second dalbavancin dose, for an additional two weeks, which would amount to six weeks of the recommended antibiotic treatment for MRSA bacteremia.

## Discussion

In light of the challenges associated with ensuring adherence to antibiotic regimens in outpatient settings, especially among populations with social barriers, such as homelessness, limited transportation, and IV drug abuse, our case report offers a possible solution. Our extensive review of literature on PubMed on the use of dalbavancin in treating MRSA bacteremia only revealed four documented cases. While three documented cases report unsuccessful results with dalbavancin in MRSA bacteremia [10-12], there is a single case indicating successful treatment [13]. Our case adds to this body of knowledge, showcasing another successful instance of treating MRSA bacteremia, notably in conjunction with MRSA osteomyelitis.

It is our opinion that the once-weekly dosing schedule of dalbavancin stands out as a critical benefit, particularly for patients who are poor candidates for frequent dosing schedules. The less frequent dosing regime of dalbavancin not only addresses the issue of adherence but also aligns with the preferences of these patients, potentially leading to improved treatment outcomes.

This case exemplifies how dalbavancin's unique dosing schedule can effectively manage complex infections like MRSA bacteremia in challenging outpatient scenarios. The upcoming DOTS (Dalbavancin as an option for treatment of S. aureus bacteremia) randomized controlled trial is expected to shed further light on its efficacy compared to standard treatments in MRSA bacteremia [14]. The outcomes of this trial could provide clinicians with the confidence to consider dalbavancin as a viable alternative, especially in cases where adherence to traditional antibiotic regimens is problematic.

## Conclusions

The once-weekly dosing of dalbavancin presents a promising solution for improving antibiotic adherence, particularly in outpatient settings and among vulnerable populations facing barriers like homelessness and limited access to transportation. While current literature offers mixed results on dalbavancin's effectiveness in MRSA bacteremia, our case adds to the growing evidence supporting its use.
